# Formulation of Microbial Inoculants by Encapsulation in Natural Polysaccharides: Focus on Beneficial Properties of Carrier Additives and Derivatives

**DOI:** 10.3389/fpls.2020.00270

**Published:** 2020-03-10

**Authors:** Nikolay Vassilev, Maria Vassileva, Vanessa Martos, Luis F. Garcia del Moral, Jolanta Kowalska, Bartosz Tylkowski, Eligio Malusá

**Affiliations:** ^1^Department of Chemical Engineering, Institute of Biotechnology, University of Granada, Granada, Spain; ^2^Department of Plant Physiology, University of Granada, Granada, Spain; ^3^Institute of Plant Protection – National Research Institute, Poznań, Poland; ^4^Chemical Technology Unit, Technology Centre of Catalonia, Tarragona, Spain; ^5^Research Institute of Horticulture, Skierniewice, Poland

**Keywords:** biofertilizers, formulation, immobilization, polysaccharides, additives

## Abstract

In the last 10–15 years, the wide application of bioformulated plant beneficial microorganisms is accepted as an effective alternative of chemical agro-products. Two main problems can be distinguished in their production and application: (a) economical competiveness based on the overall up-stream and down-stream operational costs, and (b) development of commercial products with a high soil-plant colonization potential in controlled conditions but not able to effectively mobilize soil nutrients and/or combat plant pathogens in the field. To solve the above problems, microbe-based formulations produced by immobilization methods are gaining attention as they demonstrate a large number of advantages compared to other solid and liquid formulations. This mini-review summarizes the knowledge of additional compounds that form part of the bioformulations. The additives can exert economical, price-decreasing effects as bulking agents or direct effects improving microbial survival during storage and after introduction into soil with simultaneous beneficial effects on soil and plants. In some studies, combinations of additives are used with a complex impact, which improves the overall characteristics of the final products. Special attention is paid to polysaccharide carriers and their derivates, which play stimulatory role on plants but are less studied. The mini-review also focuses on the potential difficulty in evaluating the effects of complex bio-formulations.

## Introduction

Different groups of soil microorganisms, such as root endophytic fungi, mycorrhizal fungi, plant growth-promoting rhizobacteria, rhizobia, and phosphate solubilizers affect plant growth through direct and plant-mediated mechanisms, including in stressed conditions (van der Heijden et al., 2008; Berg, 2009; Shilev et al., 2019). The application of selected plant beneficial microorganisms individually or as microbial consortia with multifunctional properties is an important tool to promote crop health and productivity (Ahmad et al., 2018; Maron et al., 2018). The scientific literature abounds in studies on isolation and characterization of plant-beneficial microorganisms, but only few of them have reached the commercial market. Many commercial bio-inoculants do not work under field conditions with the efficiency demonstrated in greenhouse or laboratory experiments (Stephens and Rask, 2000; Vassilev et al., 2015; Arora and Mishra, 2016; Malusá et al., 2016) due to inadequate and/or poor quality formulation, including poor compatibility and stability of the carriers (Bhattacharyya and Jha, 2012; Bashan et al., 2016; Baez-Rogelio et al., 2017; Stamenkovic et al., 2018).

The main roles of the formulation of inoculants are: (i) to provide a more suitable micro-environment for the microbial strain/s, combined with physical or chemical protection over a prolonged period, in order to avoid a rapid decrease of the cells’ viability during storage, (ii) to support the strain/s competition with the better-adapted native soil microflora, and (iii) to reduce losses due to the depredation by the micro-fauna after being introduced into soil. All these functions are aiming at providing a reliable source of living cells available to interact with plants and soil microbiome (Bashan, 1998; Herrmann and Lesueur, 2013; Bashan et al., 2014; Malusá and Vassilev, 2014). Indeed, a critical number of cells are essential to obtain the expected positive response from the formulated inoculum (10^6^–10^7^ cells/plant; Bashan, 1986).

Different microbial formulations have been developed using liquid or solid materials as carriers. Liquid inoculants are microbial cultures modified with water, oil or polymers (i.e., additives) that improve cell-suspension viscosity, stability and dispersion capacity (Catroux et al., 2001; Bashan et al., 2016; Malusá et al., 2016). The problem with this type of products is that the microbial population and its metabolic activity decrease rapidly after the introduction of cell suspensions into the soil, particularly if they are not containing suitable additives. A special attention has been paid in the recent years on cell-free formulations (Bashan et al., 2016) like fermentation broth filtrates (Kumar et al., 2012; Vinale et al., 2014; Vassilev et al., 2017). Since some plant beneficial microorganisms demonstrated multiple activities (Vassileva et al., 2010), their culture extracts contain various metabolic products such as antibiotics, siderophores, toxins, lytic enzymes (Thrane et al., 1997; Aydi-Ben Abdallah et al., 2014), and solubilized phosphate (Mendes et al., 2017; Vassilev et al., 2017), which positively affect the plant growth. Such type of products and the related strategy can be denominated as post-biotic.

The solid formulations are based on inorganic or organic carriers, prepared in solid, granular, or powdery forms and classified according to their particle sizes or application mode (Adholeya and Das, 2012; Malusá et al., 2012; Stamenkovic et al., 2018). The most important solid formulations are based on carriers such as peat, compost, agro-industrial wastes, vermiculite, perlite, rock phosphate, calcium sulfate, and polysaccharides (Sahu and Brahmaprakash, 2016). In the recent years, in the field of solid formulation technologies, more attention is paid to polysaccharide-immobilized inoculants (Malusá et al., 2016) as well as to inoculants produced under solid-state fermentation (SSF) conditions using agro-industrial wastes (Vassilev and Mendes, 2018). SSF processes offer many advantages including co-cultivation of two microorganisms, enrichment with soluble P (Mendes et al., 2015), induction of biocontrol activity (Vassilev et al., 2009), as well as the use of solid substrates alone, combined, and moistened with liquid wastes (Vassilev and Mendes, 2018). However, the gel-cell immobilized approach is the technological solution that can better assure a standardization of the formulated inoculum as well as its quality.

In this mini-review, we analyze the immobilized-cell approach underlying the possibilities for its improvement and some specific characteristics of the carrier structure and formulation, particularly the role of additional compounds introduced into the cell-gel structures and the effect of the gel-forming polysaccharides and their derivates on plant health and growth.

## Cell-Immobilization as a Tool for Inoculant Formulation

In bio-immobilization technology, water-soluble polymeric materials such as agar, methoxy-pectin, gellan gum, and mixtures of xanthan and locust bean gum, among many others, are largely used in the production of microbial-based products but alginate and carrageenan are the most used polymer-forming materials in microbial formulations to be introduced into soil-plant systems (Bashan, 1998; Vassilev et al., 2001, 2005, 2014). The most frequently applied method of microbial cells/spores encapsulation uses the technique of interfacial polymerization.

There are a number of review papers describing in details the advantages and the “know-how” of the immobilization technology applied in formulation of plant beneficial microorganisms (Vassilev et al., 2001, 2005, 2014, 2015; Malusá et al., 2012; Bashan et al., 2016; Stamenkovic et al., 2018). Despite obvious benefits of immobilized-cell formulations of plant beneficial microorganisms having a controlled cell-release, their large-scale production and field application are still limited. One of the main reasons is the relatively high production cost (Vassilev et al., 2001; Chen et al., 2013; Bashan et al., 2016), since the cost of the polymeric carrier is higher than the other solid and liquid formulation components (John et al., 2011). Furthermore, the structure of a polymer carrier (e.g., that of alginate) is characterized by a low mechanical strength, which determines an unstable, uncontrolled release of its content. Cell mortality during the drying of encapsulated cells has also been recognized as a critical point of the bioencapsulation process (Cassidy et al., 1996; Bashan et al., 2002).

A future frontier in this field and one of the solutions of the above problems is the development of *polymeric nanoparticle coatings* (nano-formulations) or microencapsulated formulations. Microcapsules formulated by Wu et al. (2011) enhanced the survival rate of *Klebsiella oxytoca* Rs-5 under salinity stress. The cells released from microcapsules reached up to 10^10^ cfu/g when immersed in physiological saline solution for 3 weeks, improving cotton growth under high salinity conditions in pot experiments. However, there is the need to fully evaluate environmental and health safety issues before such technology could be implemented at industrial level (Kah, 2015).

Another possibility to develop a cost-effective encapsulated formulation is to find a low-cost gel carrier or gelling agent or partly replace the expensive polymer with low-cost additives. Nano-additives might enhance the stability of microbial-encapsulated products with respect to environmental conditions (e.g., desiccation, heat and UV inactivation) or provide substances needed by the inoculum and consequentially improve the shelf-life of these products or their delivery (Jampílek and Králová, 2017; Prasad et al., 2017). Table 1 illustrates the beneficial effects of some additives.

**TABLE 1:** Examples of beneficial effect of additives on inoculant gel-based formulations.

Microorganism	Additive	Beneficial effect	References
*Pseudomonas cepacia; Talaromyces flavus; Penicillium oxalicum; Gliocladium virens; Trichoderma viride*	Pyrophyllite	Bulking agent	Fravel et al., 1985
*Pantoea agglomerans; Trichoderma harzianum*	Chitin, kaolin or bentonite	Reduced UV transmition	Zohar-Perez et al., 2003
*Raoultella planticola*	Bentonite	Continuous cell release	He et al., 2015
*P. putida*	Perlite	Cell-gel stability	Liffourrena and Lucchesi, 2018
*Azospirillum brasilense*	Skim milk	Increased cell number in beads	Bashan et al., 2002
*P. fluorescens*	Skim milk	Increased cell number and soil; enhanced cell viability	Power et al., 2011
*Enterobacter sp.*	Skim milk	Better mycorrhization	Vassileva et al., 1999
	Skim milk and montmorillonite	Higher cell survival rate	Vassilev et al., 1997
*Pseudomonas aeruginosa*	Skim milk and clay minerals	Higher plant growth promotion	Cassidy et al., 1995
*Fusarium oxysporum*	Starch	High cell viability, shelf life, and soil colonization	Bailey et al., 1998
*A. brasilense*	Starch	Extended shelf life	Ivanova et al., 2005
*Penicillium janthinellum*	Chitin and dry dry olive wastes	Chitinase synthesis; biocontrol activity	Vassilev et al., 2008
*Bacillus subtilis*	Humic acids	Higher survival rate	Young et al., 2006
*Raoultella terrigena*	Trehalose	Desiccation protection	Schoebitz et al., 2013
*Bacillus salmalaya*	Protein hydrolysate	High encapsulation index	Vejan et al., 2018

## The Role of Additives on the Overall Performance of Immobilized Inoculants

### Clay Minerals

There is a wide selection of additional materials used in bio-immobilized systems, which can serve as carrier bulking agents, enhance the formulation stability, protect and feed microbial cells or spores. Since the early studies on gel-entrapped soil microbial inoculants, polysaccharides/clay minerals combinations were used to protect the immobilized/encapsulated cells and to ensure their slow release into the environment (Marshall, 1968; Jung et al., 1982; van Elsas et al., 1992; Vassileva et al., 1999; Bashan et al., 2002). *Clay minerals* such as pyrophyllite have been experimented as bulking agents (Fravel et al., 1985) and bentonite and kaolin were used as fillers in alginate-glycerol immobilized *Pantoea agglomerans* and *Trichoderma harzianum* (Zohar-Perez et al., 2003). The freeze-dried alginate-bentonite and alginate-kaolin combinations had a considerable positive effect on the bead’s average wall thickness and significantly increased microbial survival reducing UV transmission compared to free-cell and cells immobilized in alginate-glycerol without fillers.

The addition of bentonite to alginate-based formulation was found to increase the solid content and the porosity of alginate polymer used as a carrier of *Raoultella planticola* (He et al., 2015). Without bentonite, the release of the immobilized microbial cells was rapid in the first 3-day period followed by a constant cell release, while the presence of the additive regulated the continuous flow of the microorganism to the soil. *Pseudomonas putida* Rs-198 microencapsulated with a mix of alginate, bentonite and starch was reported to increase cotton biomass, soluble protein content, and chlorophylls a, b and carotenoid concentrations of cotton grown under saline conditions (He et al., 2017).

Liffourrena and Lucchesi (2018) applied perlite as filler of alginate microbeads formed in CaCl_2_ – paraffin emulsion mixture to formulate *P. putida* biostimulant. The number of cells reached 10^8^ CFU/g micro-beads and the increase in cell-gel mechanical stability was proportional to perlite concentration. This amount was sufficient to colonize *Arabidopsis thaliana* rhizosphere, with an increase in colonization over time from 2.1 × 10^4^ to 9.2 × 10^5^ CFU/g soil after 21 days.

### Skim Milk

Skim milk is another additive widely used in bioformulations to enhance cell viability after storage (Yu et al., 2001). Bashan et al. (2002) found that the addition of skim milk powder to alginate-encapsulated *Azospirillum brasilense* significantly increased the cell number within the cell-bead structure. These beads degraded faster in soil than beads without skim milk thus releasing rapidly the entrapped cells into the soil-plant system. Alginate carrier with 10% skim milk significantly increased the numbers of *Pseudomonas fluorescens* cells released into the soil compared to combinations with soil extract and control beads (Power et al., 2011). After 250 days of storage, 100% recovery of viable cells was obtained from skim milk-alginate encapsulated *P. fluorescens*. *Enterobacter* sp. encapsulated in alginate gel enriched with 3% skim milk stimulated plant mycorrhization and demonstrated better bacterial establishment and phosphate-solubilizing activity in soil (Vassileva et al., 1999). This resulted in both higher growth of *Lactuca sativa* and higher number of cells released in the soil in comparison with plants inoculated with formulations without skim milk.

Designing complex formulations containing both skim milk and clay materials can be a strategy to increase the inoculum efficacy in comparison to single additives. Bentonite clay (3% w/v) was found to increase the positive effect of skim milk powder (3% w/v) on the survival rate of *P. fluorescens* R2f encapsulated in alginate (van Elsas et al., 1992). In another study, with *lac-lux* marked *P. aeruginosa*, a 1% κ-carrageenan amended with skim milk and bentonite:montmorillonite (60:40%) was more effective compared to alginate-skim milk formulation particularly after a 3-month storage of the dried beads (Cassidy et al., 1995). Vassilev et al. (1997) used skimmed milk and clay as additives to enhance metabolic activity in fermentation and soil conditions as well as the survival rate of the P-solubilizing *Enterobacter* sp. entrapped in agar beads.

### Starch

Starch has been well studied in various biotechnological schemes with dried beads or liquid core capsules (Jankowski et al., 1997; Kim et al., 2005). It has successfully been used as a carrier or additive in formulations of plant beneficial microorganisms. In the bioencapsulation matrix, starch reduced the physical stress to microbial cells and significantly improved their survival (Bashan et al., 2002). *Bacillus thuringiensis* was entrapped in gelatinized corn starch (Shasha et al., 1984), which coupled with broad-band UV screens such as Congo red provided protection from solar radiation (Dunkle and Shasha, 1989). The effectiveness of this system in conidia formation per gram of mycelium was confirmed with the entomopathogenic fungi *Metarhizium anisopliae* and *Beauveria bassiana* (Pereira and Roberts, 1991). Complex formulations based on alginate-starch were used to formulate myco-herbicidal strains of *Fusarium oxysporum* (Bailey et al., 1998), which showed high viability/shelf-life and rhizosphere colonization rate. Two endophytic fungi (*Muscodor albus* and *Muscodor roseus*) producing volatile myco-fumigants were formulated in a mixture of water-absorbent starch, corn oil, sucrose, and fumed silica (Stinson et al., 2003). The produced formulations reduced the disease incidence of soilborne pathogens but plant growth reduction was observed probably due to the growth of deleterious rhizobacteria on some components of the complex carrier.

The protective effect of starch on the microbial cells under stress conditions is based on the cell adhesion to the starch. This process depends on the strain and the relationship between the adhesion to the starch and its use as a substrate (Crittenden et al., 2001). Furthermore, Tal et al. (1999) reported that the strength of an alginate-starch bead is directly proportional to its starch content and the distribution of starch granules within the beads is homogeneous at higher starch concentration. Even though the porosity of the beads’ structure decreases with an increase in their starch content, the opposite tendency is observed after a period of storage when the porosity increases as the immobilized cells utilize the starch. The later phenomenon resulted in bacterial population levels of up to 10^9^ CFU/bead in dry alginate-starch beads (Ivanova et al., 2005).

### Chitin and Chitosan

Chitin and chitosan are oligosaccharides used in formulations as fillers or coating material, respectively. Chitosan is a bioactive polymer with a wide variety of functional properties such as antibacterial activity, non-toxicity, ease of modification, and biodegradability (Muxika et al., 2017). Addition of chitin or chitin-containing materials improved the multiplication of *Bacillus subtilis* and its fungicidal activity to control Fusarium wilt (Manjula and Podile, 2001). Chitin and dry olive wastes (DOW) were mixed with alginate to encapsulate *Penicillium janthinellum* (Vassilev et al., 2008). The fungus showed higher chitinase synthesis compared to alginate-entrapped mycelium, even when this was added singly with DOW or chitin. The three-component formulation induced P-solubilizing fungal activity while alginate-chitin formulation exhibited biocontrol activity suppressing the soil-borne pathogen *F. oxysporum*. The use of chitin/chitosan to encapsulate microbes can also ease the storing and application on farms, which has been one of the major restriction to the use of biopesticides in recent times (John et al., 2011).

Chitosan is an excellent chelating agent, well known for its biocontrol activity against pathogens (Goy et al., 2009; Franco and Peter, 2011; Berger et al., 2014) and as elicitor enhances stress tolerance, antioxidant activity, and production of osmoregulators in plants (Dar et al., 2015). As a coating material, chitosan can lower the formulation cost, making the final product multifunctional due to its biocontrol and plant strengthening activities. The formulations can be produced by dropping alginate in a chitosan-CaCl_2_ solution or introducing already formed alginate beads into chitosan solution (Wittaya-Areekul et al., 2006).

Chitosan can also be an excellent carrier for plant beneficial microorganisms (Chanratana et al., 2018). Applying the methodology used for the development of a controlled-release fertilizer (Perez and Francois, 2016), where starch was added to a chitosan-based formulation as a filler, using a sodium tripolyphosphate aqueous solution as the crosslinking agent, *A. brasilense* and *P. fluorescens* were encapsulated in chitosan-starch formulation (Perez et al., 2018). The formulated bacteria survived at least 12 months at room temperature and humidity, maintaining a high viability (10^9^ CFU of *A. brasilense*/g and 10^8^ CFU of *P. fluorescens*/g). When introduced in soil, the bacterial cell number increased progressively during the first 20 days and then decreased.

### Humic Acids

Humic products are known to promote or decrease the populations or activities of specific microbiome species (Pukalchik et al., 2019). Encapsulation of *B. subtilis* in alginate beads supplemented with humic acids ensured high viability of the immobilized biostimulant (Young et al., 2006). The immobilized gel-humic acid-cell system demonstrated excellent survival rate after storage for 5 months and slow cell release at various levels of pH, providing also successful plant growth promotion by the encapsulated bacteria. The positive effect of this additive on plant growth can be explained considering its role as stimulant of the microbial growth and activity (Rekha et al., 2007) as well as for its effects on the physiology of plants (Nardi et al., 2002). The addition of humic acids in bacteria formulations could be also useful to promote root colonization by native mycorrhizal fungi (Gryndler et al., 2005).

### Sugars

Sugars, such as sucrose, trehalose or glucose, are widely used to preserve microorganisms from changes in the osmotic pressure and can contribute to their conservation and maintenance particularly after drying (Morgan et al., 2006). However, combinations between sugars and sugars-additives have not been studied before and after the formulation of microbial-based products although various types of sugars and other additives (see previous paragraphs) usually improve the overall encapsulation efficacy. The addition of trehalose to the growth medium increased the survival of *Raoultella terrigena* during the drying process much more effectively protecting against desiccation than adding it to the matrix solution just prior to drying (Schoebitz et al., 2013). The complex formulation of *B. bassiana* based on the use of skimmed milk powder, polyvinylpyrrolidone K-90, and glucose was reported to achieve 100% conidial germination and 78% conidial viability, even after storage for 12 months at 30°C (Mishra et al., 2013).

### Protein Hydrolysates

Protein hydrolysates derived from animal wastes and plant biomass after chemical, thermal and enzymatic hydrolysis, have been shown to enhance both nutrient uptake by plants and soil microbial activity (Colla et al., 2017; Casadesús et al., 2019). The latter was suggested to be the result of the stimulating presence of the organic molecules in protein hydrolysates, which serve as nutrients for the rhizospheric and phyllospheric microorganisms (Colla et al., 2017). Vegetable protein hydrolysates received more research interest particularly as co-polymers in microcapsules in the food, pharmaceutical and cosmetics industries (Nesterenko et al., 2013) but also in biostimulant production (Colla et al., 2015). In a recent work, *B. salmalaya* was encapsulated in chitosan-alginate-protein (brown rice) capsules, formulated in slurry or powder achieving an encapsulation index of 99.7 and 89.3%, respectively (Vejan et al., 2018). Such result underlines the importance of additives based on vegetable proteins in future studies on formulation of biostimulants by gel-encapsulation.

### Glycerol, Silicon, Poly-Lactic Acid, and Strigolactones

Some compounds with well-manifested functions advantageous to microorganisms could also be considered as potential additives. In a recent article we have analyzed the potential of *glycerol*, a trihydroxyalcohol widely used as a cell viability protector in strains’ maintenance practice, in this respect and suggested the need for more studies on its application in formulation techniques (Vassilev et al., 2017). Similarly, *silicon* has not found wide applications as biostimulant to plants yet, particularly in encapsulated-cell formulations, although its benefits were widely reviewed (Savvas and Ntatsi, 2015). Trials using hydrophobic silica nanoparticles to the water-in-oil emulsion have shown an improvement in the delivery of the product, as well as an enhancement in shelf life by reduction of desiccation (Kaushik and Djiwanti, 2017). The use of new polymer-forming materials such as *poly-lactic acid* (PLA) could also open new possibilities to develop encapsulated inocula that would benefit of the physical characteristics of these compounds (Lai et al., 2009). *Strigolactones* (synthetic analogs), which communicate with the plant-microbiota systems, have been suggested as potential active additives (Vassilev et al., 2015) but as they demonstrate stimulating signals to parasitic plants and microorganisms (De Cuyper and Goormachtig, 2017), it would be challenging to develop an effective complex gel-based biostimulants.

## Potential Effect of the Gel-Forming Polysaccharides on Plant Health and Growth

Encapsulation of inoculant cells in polymers of polysaccharides such as alginate and carrageenan has been proposed long time ago as a technique to ensure controlled release of plant beneficial microorganisms into soil (Dommergues et al., 1979; Bashan, 1986). Surprisingly, few studies have examined simultaneously the fate of the gels in soil and the effect of the cell-free carriers on plants and rhizosphere microbiota. The positive effect on plants of seaweed crude extracts, the raw material from which several polysaccharides used for encapsulation technology derive, is based on the synergic action of growth regulators, osmolytes, polysaccharides and other algal compounds (Battacharyya et al., 2015). Seaweeds are known for their action as bioelicitors and particularly laminarin, carrageenan, and alginate, have been studied for their plant defense stimulating effects (Chandía et al., 2004; Khan et al., 2009; El Modafar et al., 2012; Vera et al., 2012; Zhang et al., 2015; Abouraicha et al., 2017; Ben Salah et al., 2018). There are strong evidences that polysaccharides play an important role in the mechanisms of abiotic stress protection for microorganisms (Vassilev et al., 2012). The production of alginate as exopolysaccharide increased in bacteria growing under drought conditions (Sa et al., 2019) creating a hydrated microenvironment contributing to biofilm architecture (Chang et al., 2007).

Particularly attractive for increasing plant growth and health are oligosaccharides derived from natural polysaccharides as they play the role of signal molecules regulating plant development and defense (Larskaya and Gorshkova, 2015). They can be obtained by enzymatic (Murata et al., 1993) and acidic depolymerization (Haug et al., 1966), and thermal polysaccharide treatment (Aida et al., 2010). Oligosaccharides produced by different methods demonstrated different physiological activities in animal cells (Iwamoto et al., 2005), but this phenomenon has not been widely studied on soil-plant-microbiota systems. A great part of studies on the plant growth promoting effect of oligosaccharides are performed after γ-irradiation of polysaccharides such as chitosan, κ-carrageenan and alginate. In this latter case, the effect of the resulting products on plants was higher when the irradiation was performed in solid-state compared to liquid solution (Hien et al., 2012). Oligochitosan, obtained after gamma-irradiation, was defined as a growth stimulator and anti-microbial agent for various plant systems (Muley et al., 2019a) including under conditions of drought stress (Muley et al., 2019b). Oligosaccharides (galacto-, isomalto-, fructo-, and xylo-) used as a part of alginate gel beads were reported to enhance cell viability of oral *Lactobacillus fermentum* and carrier stability when exposed to the specific environmental conditions (Liao et al., 2019). Similar inclusion of oligosaccharides should be expected in the near future in formulations of plant biostimulants. Other studies should be carried out on the behavior of the polysaccharide carriers in soil-plant systems, particularly to unravel in more details their degradation processes in soil by plants and/or microorganisms producing polysaccharide-cleaving enzymes. Such studies could help addressing the question on how the soil microbiota and plants are affected by the polysaccharide derivates, including oligosaccharides, composing the formulation of microbial-based products.

## Conclusion

Significant progress has been made in developing formulations of plant beneficial microorganisms by entrapment in natural water-soluble polymer-based carriers and their application as biostimulants. However, published reports often do not consider or discuss the changes in the carrier characteristics by the entrapped cells or additives. There are indications that changed properties of polymers in presence of additives positively affect their ability to maintain and protect the microorganisms. Moreover, in many cases the additives potentially affect plant growth and health and simultaneously induce microorganisms to release metabolites thus provoking changes in the typical gel structure and integrity. The resulting microbial and gel side-products might stimulate plant growth and exert biocontrol activity. In some cases, it has been shown that additives are exerting a negative effect on the whole cell encapsulation system. Manitol was reported to decrease germination of gel-encapsulated spores (Liu et al., 2015). Viveganandan and Jauhri (2000) found that charcoal-soil, mixed with alginate, adversely affected the loading and survival of phosphate-solubilizing bacteria. Therefore, a deeper analysis of the relationship carriers-additives-microorganisms-soil-plant systems can provide important information that is essential to understand the functional characteristics of immobilized biostimulants and determine strategies for their application. Research efforts should also be oriented toward development of micro-environmental conditions to facilitate the growth and functional activity of the bioformulates, including in the carriers specific prebiotic compounds. As we repeatedly pointed out, research scientists working with immobilization methods should use techniques already proven in other biotechnological fields. Further improvement of immobilized cell methodologies should be based on multidisciplinary research of wide number of experts in microbiology, plant physiology/pathology, formulation specialists and agricultural engineers in order to provide efficient, safe, economically acceptable, and easy to apply complex biotechnological products for plant growth and health.

## Author Contributions

NV and MV designed and drafted the work. EM, LG, VM, JK, and BT contributed to the revision of the manuscript.

## Conflict of Interest

The authors declare that the research was conducted in the absence of any commercial or financial relationships that could be construed as a potential conflict of interest.
